# Concept and application of relaxing radial retinectomy for retinal detachment with advanced proliferative vitreo-retinopathy

**DOI:** 10.1186/s40942-020-00251-y

**Published:** 2020-10-01

**Authors:** Waldensius Girsang, Dwi C. R. Sari, Wahyu Srigutomo, Tjahjono D. Gondhowiardjo, Muhammad B. Sasongko

**Affiliations:** 1 Jakarta Eye Center Eye Hospitals and Clinics, Jakarta, Indonesia; 2 Department of Ophthalmology, Faculty of Medicine, Public Health and Nursing, Universitas Gadjah Mada - Sardjito Eye Center, Dr. Sardjito General Hospital, Jalan Farmako Sekip Utara, Yogyakarta, Indonesia; 3 Department of Anatomy, Faculty of Medicine, Public Health and Nursing, Universitas Gadjah Mada, Yogyakarta, Indonesia; 4 Faculty of Mathematics and Natural Sciences, Bandung Institute of Technology, Bandung, Indonesia; 5 Department of Ophthalmology, Faculty of Medicine, Universitas Indonesia/Cipto Mangunkusumo National Hospital, Jakarta, Indonesia

**Keywords:** Rhegmatogenous retinal detachment, Proliferative vitreoretinopathy, Radial retinectomy, Vitrectomy, Relaxing retinectomy

## Abstract

**Purpose:**

To revisit the concept of retinectomy and the theory of mechanical forces on the retina occurring in rhegmatogenous retinal detachment (RRD) and to describe the potential application of radial retinectomy in RRD with advanced proliferative vitreoretinopathy (PVR).

**Methods:**

A literature search was performed to identify all English language articles reporting the use of retinectomy for the management of RRD with PVR. We reviewed the theoretical background of mechanical forces occurring in RRD.

**Results:**

Detachment of the retina from the retinal pigment epithelium (RPE)/choroid is influenced by disequilibrium of several physical forces: tangential forces on the epiretinal membrane $\left( {T_{1} } \right)$ and radial traction on the retina $F_{R}$ exceeding the retinal adhesion force to the RPE $\left( {T_{1} \;\text{ + }F_{R} \;\text{ > }\;F_{A} } \right)\,\,$. PVR may exaggerate the amounts of tangential and radial forces ($\left( {T_{1} } \right)$ and $F_{R}$) that pull the retina off. Relaxing radial retinectomy, by the nature of its cutting pattern, may theoretically decrease the amounts of both forces, therefore restoring the equilibrium between tensile and adhesive forces on the retinal surface $\left( {T_{1} \;\text{ + }F_{R} \;\text{ = }\;F_{A} } \right)\,\,$.

**Conclusion:**

Relaxing radial retinectomy may potentially be applied in RRD with advanced PVR but has rarely been reported to date. Future studies are needed to evaluate its outcomes and long-term complications.

## Background

Rhegmatogenous retinal detachment (RRD) is the most common form of retinal detachment [1] and is characterized by the presence of a retinal break as an entry point of fluid into the subretinal space, leading to separation of the neurosensory retina (NSR) from the retinal pigment epithelium (RPE) [2, 3]. Management of RRD is considered very routine for vitreoretinal (VR) surgeons but can be very challenging when the condition progresses with the presence of proliferative vitreoretinopathy (PVR) [4, 5]. PVR is an abnormal cellular accumulation generating traction on the retina that may significantly reduce the anatomical success rate of RRD treatment due to the double burden and difficulty level of the surgery [5, 6].

Theoretically, in retinal detachment, mechanical forces caused by PVR play a key role in generating traction and ultimately reduce the success rate of retinal reattachment surgery [7]. Therefore, any procedures performed during surgical treatment of RRD with PVR should be targeted at reducing or eliminating these forces to achieve anatomical reattachment of the retina. Retinectomy, introduced in 1979, is a technique to partially incise the retina with the intent to reduce tractional forces at the retina for the management of RRD with advanced PVR [8]. Subsequently, variations of retinectomy techniques, such as circumferential, anterior flap and radial retinectomy, were developed with the aim of improving anatomical outcomes of RRD with advanced PVR [9–12]. However, no reports have discussed these techniques from the theoretical perspective in greater detail.

In this review, we aimed to revisit the concept of retinectomy and the theory of mechanical forces on the retina during retinal detachment and to propose radial retinectomy as a potential approach specifically in the context of retinal detachment with advanced PVR.

## Rhegmatogenous retinal detachment

RRD is the most common form of retinal detachment. RRD occurs in association with a full-thickness retinal break or tear [3]. The outset of RRD is vitreous liquefaction, known as vitreous syneresis [2, 6]. Vitreous syneresis usually results naturally due to aging but can be accelerated by several pathologic processes, such as high myopia, cataract surgery, or ocular trauma [13]. Significant vitreous syneresis further causes posterior vitreous detachment (PVD), an acute event that generates tractional forces [2, 3]. These tractional forces are transmitted to areas with persistent or strong vitreous attachment. If these forces and vitreous attachment are strong, the retina breaks. During PVD, retinal breaks may develop anywhere in the retina with or without firm vitreous adhesion [14, 15]. When a retinal break occurs, ocular movement may cause liquified vitreous fluid to exude into the subretinal space and accumulate between the NSR and RPE [2, 3].

## Development of proliferative vitreoretinopathy

PVR has attracted substantial attention over the last two decades for many VR surgeons because it may determine the complexity of the surgery and may also significantly influence the success rate of RRD repair [4]. PVR is an outcome of ectopic cell proliferation (retinal pigment epithelial cells and glial cells) in the vitreous and/or periretinal area, resulting in periretinal membrane formation (epiretinal and subretinal), in turn causing retinal traction [4, 5]. Currently, accumulating evidence in this area has contributed to a more complex understanding of the pathophysiology of PVR. Retinal breaks, instantaneous separation of the NSR and RPE and the subsequent hypoxia in RRD may trigger tissue trauma, which initiates inflammation and wound healing responses similar to those in tissues elsewhere in the body [4, 5, 16]. Various cells in the retina—e.g., retinal pigment epithelial cells, glia and fibroblasts—participate in this pathological wound healing response, leading to active tissue remodeling and scar formation [4, 5, 16].

Mechanical stress at the site of RRD stimulates glial cells to release proliferation factors, causing macrophage and fibroblast proliferation and migration into the vitreous at this site [17, 18]. Retinal pigment epithelial cells migrate via retinal break and accumulate in vitreous causing a “tobacco dust” appearance. This is usually referred to as grade A PVR. Meanwhile, glial cells migrate to dedifferentiate and transform into myofibrocytes. Myofibrocytes adhere to fibrin, elastin and fibronectin released by the damaged blood-retinal barrier. This group of cells may form a membrane at the retinal surface, which typically causes the retina to wrinkle and the edge of the break to appear irregular and rolled. This condition is referred to as grade B PVR. As wound healing continues, fibroblasts contract, and the retinal membrane undergoes continuing maturation and becomes denser and more compact, resulting in rigid retinal folds and vitreous strands (grade C PVR) [4, 5, 18].

## Brief history and previous concept of retinectomy in retinal detachment surgery

The ultimate goal of RRD surgery is to restore the retina to its original position to preserve optimal macular function and to prevent subsequent redetachment as a consequence of repeated proliferation [2]. In the presence of severe PVR, the management of RRD demands more complex procedures to relieve the presenting traction. In some situations where retinal traction persists, it is reasonable to consider performing retinectomy to remove the traction. This procedure has been described previously; its primary intent is to restore the retina to its normal position, thus resulting in visual improvement [8, 9, 19].

The concept of retinotomy was first described in 1979 by Machemer, who pioneered this method to manage the treatment of eyes with retinal incarceration due to traumatic scleral wounds [8]. Živojnović and colleagues later became the first to report the use of retinectomy for the management of PVR membrane and retinal traction in RRD [19]. Subsequently, many surgeons and researchers began to report the use and outcomes of retinotomy and retinectomy for the treatment of RRD with severe PVR. Table 1 summarizes previously published studies applying different techniques of retinotomy/retinectomy, with each individual success rate ranging from 58% to nearly 100% for the management of RRD with complex PVR of at least grade C. The majority of surgeons reported the use of circumferential retinotomy/retinectomy, only a few have used combined radial and circumferential retinectomy, and none have applied radial retinectomy alone [11, 20–22].

## The concept of relaxing radial retinectomy for retinal detachment with proliferative vitreoretinopathy

### Mechanical forces on the retina occurring in retinal detachment

**Fig. 1:**
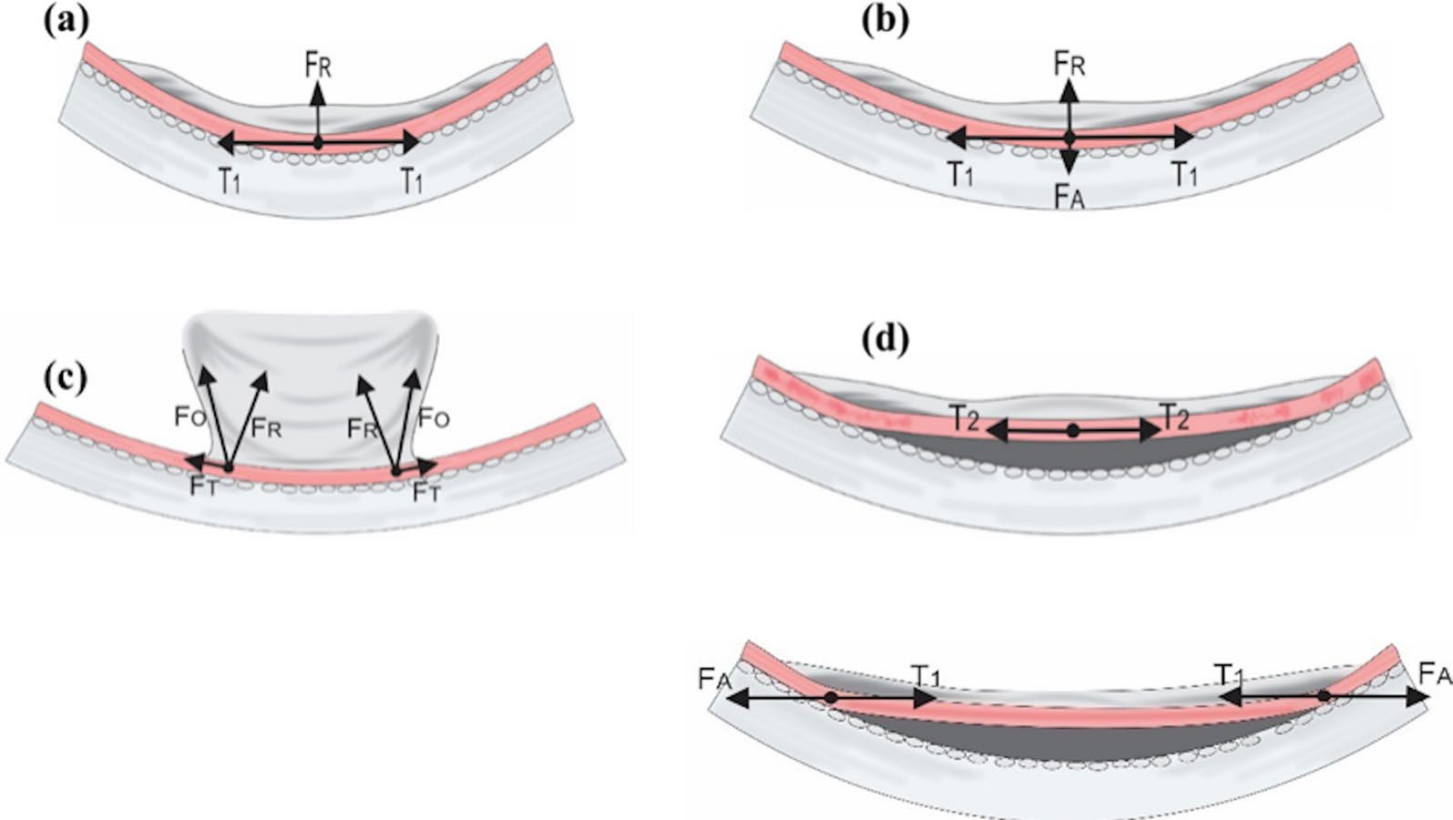
Mechanical forces on the retina

Detachment of the retina from the RPE/choroid is influenced by several physical forces: retinal tensile force, retinal adhesive force, and intraocular fluid flow [23, 24]. Figure 1 illustrates several physical forces occurring in normal and detached retinas. Physical forces on the retina have an amplitude and a direction, shown as vectors. Vectors of vitreous tensile forces towards the retina can be tangential or oblique depending on the contour and circularity of the retinal surface.

In normal eyes (Fig. 1a), the elastic force from the adhesion of the epiretinal membrane to the retinal surface that produces tangential traction ($T_{1}$) and radial traction ($F_{R}$) on the epiretinal membrane resulting from the normal circularity of the retinal surface [24–28]. In addition, a retinal adhesive force $(F_{A}$) is generated by complex interactions between oncotic fluid pressure, interphotoreceptor matrix and some metabolic factors in the retina [29, 30]. This force is equivalent to the radial traction $\left( {F_{R} } \right)$ that pulls the retina off (Fig. 1b). Retinal detachment may occur when the sum of the tangential forces on the epiretinal membrane ($T_{1}$) and the radial traction on the retina ($F_{R}$) exceed the retinal adhesion force towards the RPE $\left( {T_{1} \;\text{ + }F_{R} \;\text{ > }\;F_{A} } \right)\,\,$, or when the resultant tangential force $\left( {F_{T} } \right)$) and oblique force $\left( {F_{o} } \right)$ are greater than the adhesive force $\left( {F_{A} } \right)$ (Fig. 1c). Once the retina is detached, the tangential traction on the retina may be exerted in the opposite direction at the site of retinal detachment ($T_{2}$), shifting the direction of retinal adhesive forces ($\left( {F_{A} } \right)$ at the point where the retina remains attached to counterbalance these tangential forces (Fig. 1d) [24, 25, 27, 31].

### Application of relaxing radial retinectomy

**Fig. 2:**
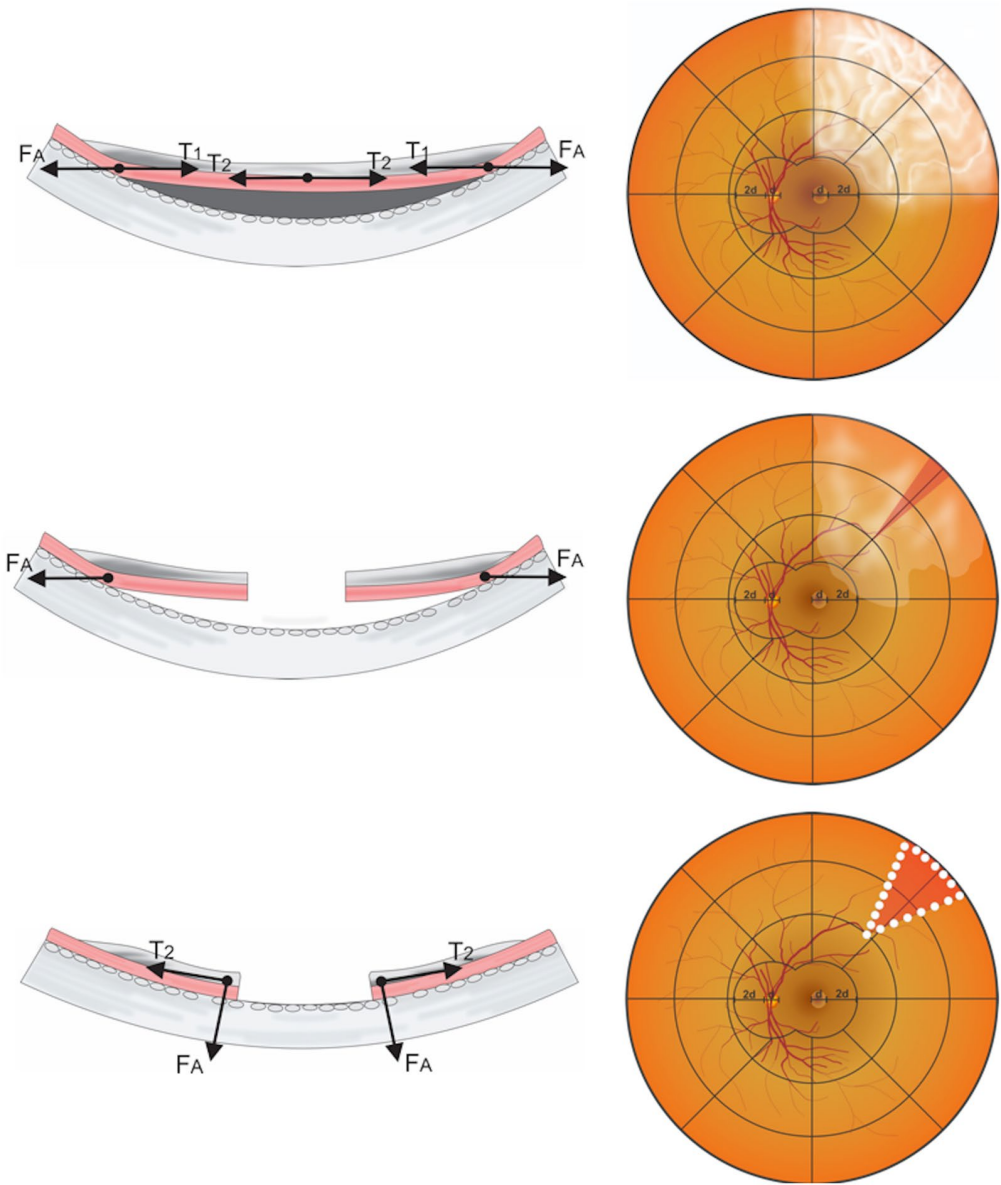
Mechanical forces on the retina after retinectomy

In principle, all approaches performed during retinal detachment surgery (e.g. vitrectomy, subretinal fluid drainage, and intraocular tamponade) aim to reduce the radial traction $\left( {F_{R} } \right)$ and tangential forces ($T_{1}$ and $T_{2}$) and optimize the retinal adhesive force $\left( {F_{A} } \right)$ [32]. The nature of radial force on the retinal surface is that it works in keeping with the circularity of the concave retinal surface [33]. Intraocular tamponade may dispense additional pressure on the retina, thus counteracting the radial and tangential forces, amplifying the strength of adhesive force and substantially increasing the success rate of the surgery [31]. However, this approach might not be entirely applicable in the presence of PVR. PVR stiffens the retina due to its membranous proliferation, and therefore may exaggerate both tangential forces ($T_{1}$ and $T_{2}$) and radial forces on the surface of the retina [7, 34]. The application of retinectomy can be considered to remove the PVR or retinal tissue, causing traction on the retinal surface [35]. While circumferential retinectomy has been a more common retinectomy technique over the past few decades, because of its cutting pattern, it reduces only the radial forces on the retina [32, 33, 36]. Radial retinectomy, by contrast, when applied across the site where PVR is promiment, may theoretically decrease the amounts of both the tangential and radial forces that counteract the retinal adhesive forces, therefore reinstating the equilibrium between these forces on the retinal surface (Fig. 2) [34].

Early surgical steps in closed posterior vitrectomy, including wound construction, vitrectomy, sub-retinal fluid drainage and removal of any existing epiretinal membrane, are generally routine procedures. Thorough retinal observation should be performed to determine the locations of retinal breaks and PVR extension before vitreous removal. In the presence of an epiretinal membrane or scar tissue, peeling or removal of the scar tissue causing retinal wrinkling is preferrably performed using forceps to lift the membranous edge. Frequently, complete retinal reattachment can be achieved after removal of scar tissue on the retinal surface without the need for retinectomy. However, in situations where retinal reattachment cannot be achieved and retinectomy is required, it is very important to carefully determine the sites for retinectomy in order to maximize preservation of the optic nerve and macula as much as possible. Anatomically, the density of photoreceptors peaks at the posterior pole, which is approximately 4.5–6 mm around the fovea centralis [37–39]. Therefore, the cutting boundaries of the retinectomy are defined considering this topographic organization of the macula and should be located more than 6 mm away from the central fovea or outside the large vascular arcade surrounding the posterior pole. In the nasal area, the closest cutting boundaries should also be located 4.5 – 6 mm from the centre of the optic nerve [37–39].

Certain situations demand careful attention. First, in the presence of extensive cellular proliferation at the vitreous base, meticulous vitrectomy and membrane peeling must be performed. Radial retinectomy should be executed in the most heavily wrinkled area of the retina, because this area has the greatest tangential traction. Second, it is also important to note that in the case of thick circumferential membrane proliferation at vitreous base with strong adhesion to the retina, the peeling technique alone may not adequately remove the membrane and eliminate traction. This situation may require a radial incision in the retina across the circumferential membrane formation to relax the traction. These aforementioned techniques would induce relaxation of the retina and flatten the retina during fluid-air exchange, especially in the peripheral retina, anatomically the thinnest point. Importantly, neither of these techniques induce retinal dialysis or avulsion. Third, when further retinal thickening and shortening is also present (grade D PVR), combination of these techniques with an external encircling buckle or circumferential retinectomy can be considered to allow sufficient elimination of traction. However, a detailed technique combining radial and circumferential retinectomy is beyond the scope of this review, and thus will not be discussed.

**Fig. 3:**
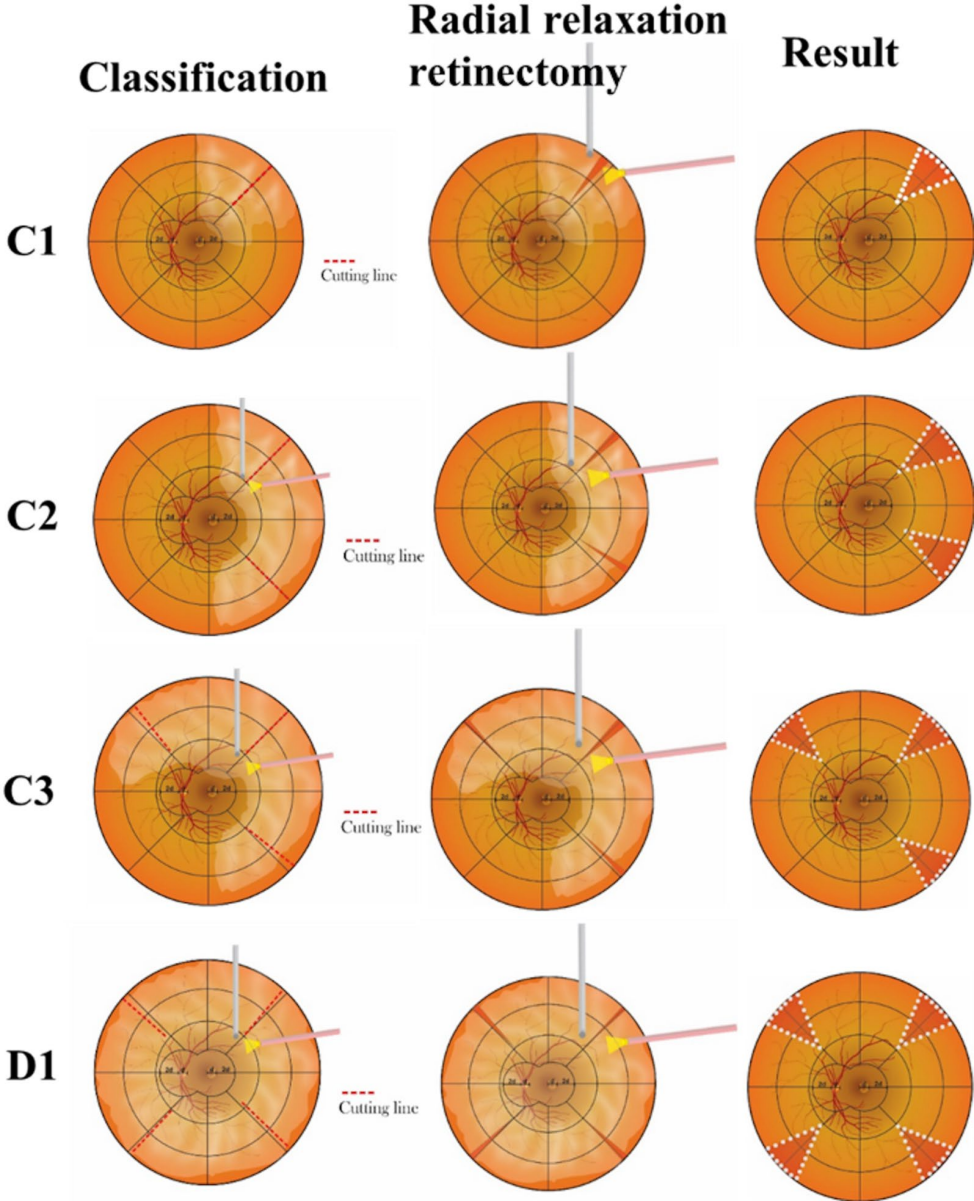
Schematic illustration of radial retinectomy for different grades of proliferative vitreoretinopathy (PVR)

Relaxing radial retinectomy is performed considering the size or grade of the PVR and should target the area of fibrotic tissue or the site of thick PVR. Retinectomy can be applied one or two times depending on the extent of the PVR. In grade C1 PVR, radial relaxing retinectomy can be performed in 1 quadrant, whereas grade C2 or C3 PVR may require two or three quadrants (Fig. 3).

## Case report

We present a case of RRD with grade C3 PVR in a patient who underwent radial relaxing retinectomy as described in the previous sections.

### Case illustration

**Fig. 4:**
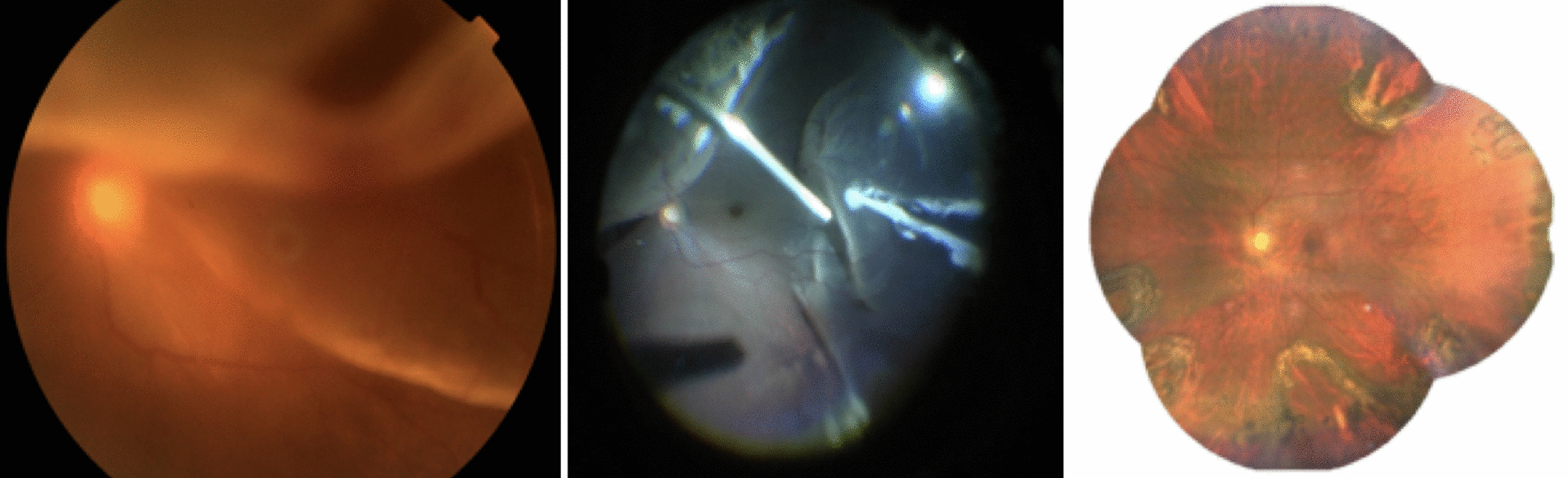
A case example of subtotal retinal detachment with grade C3 PVR

A healthy 55-year-old man experienced an abrupt change in his vision 10 weeks prior to his visit. His best corrected visual acuity (BCVA) at the time of his first visit was 1/300 with an intraocular pressure (IOP) of 8 mmHg. The patient had no signs or history of uveitis, posterior abnormalities or diabetic retinopathy with or without posterior laser treatment. General eye examination showed normal anterior segment. Posterior segment examination showed subtotal retinal detachment in all quadrants involving the central macula, with full-thickness fixed retinal wrinkle between the 11 to 8 o’clock positions, mature and immature membranes, and subretinal fibrosis extending peripherally between the 4 and 8 o’clock positions at the vitreous base. We performed vitrectomy, membrane peeling and subretinal fibrosis removal. We could not entirely remove subretinal fibrosis due to its very strong adhesion to the retina and the retina could not be flattened during fluid-air exchange; therefore, we performed radial retinectomy at three sites: one at the 1 o’clock position superior and two at the 5 and 8 o’clock inferior across the remaining region of subretinal fibrosis (Fig. 4). Additionally, a 12% C3F8 tamponade was inserted. During follow-up, the patient’s BCVA remained at 1/300 after 4 weeks but improved to 6/18 and 6/15 at 3 and 6 months, respectively (Fig. 4). The IOP measurements were 9 and 12 mmHg at his 3- and 6-month visits.

## Special consideration

As outlined in Table 1, all previously published studies reported a considerable proportion of complications within some period after RRD surgery with retinotomy/retinectomy. During retinotomy/retinectomy, the entire retina, excluding its RPE, is removed. Direct exposure of intravitreal fluid to retinal pigment epithelial cells, which are responsible for controlling the osmotic gradient of fluid transfer, increases the rate of uveoscleral outflow, resulting in ocular hypotony [40]. The possibility of ocular hypotony is significantly greater with the practice of extensive retinal laser treatment to induce chorioretinal scarring at the edge of the retinectomy [40]. More importantly, laser-induced chorioretinal scarring may also produce ischemia in the peripheral retina and choroid, which may consequently trigger neovascular glaucoma, as frequently reported in previous studies (Table 1).

While relaxing radial retinectomy is less aggressive than circumferential retinectomy and has a smaller incised area of the retina, the potential complications that may occur following this particular technique remain less certain. Therefore, caution regarding all possible complications after retinectomy is very important, and surgeons should consider performing retinectomy as the last available option after all approaches have proven unsuccessful in flattening the retina to its original position.

## Conclusions

Treatment of RRD with PVR may be considered routine among VR surgeons, yet surgical management of this condition remains challenging because postoperative anatomical results can be unsatisfactory. Circumferential retinotomy/retinectomy, introduced few decades ago, has been widely used as one of important surgical approaches for RRD surgery with complex PVR and, more importantly, has greatly improved both clinical and most notably the anatomical outcome of the surgery, despite the potential occurrence of several complications. Relaxing radial retinectomy is another retinotomy/retinectomy technique that has not been widely applied or at least has been seldom reported to date, despite its strong theoretical foundation for application in RRD with severe PVR. Whether this technique can be a substitute for previous circumferential retinectomy approaches remains to be confirmed. Future studies are needed to determine the applicability and repeatability of relaxing radial retinectomy among VR surgeons and to evaluate its outcomes in various cases and possibly its comparability with previous circumferential retinectomy. Finally, regardless of whether relaxing radial retinectomy is superior or inferior to previously established retinectomy techniques, it may offer a good addition to a surgoen’s library, which eventually enrich the treatment options for different cases, after careful consideration of potential risks and benefits.

## Methods of literature search

A literature search of English language articles was performed in PubMed, Google Scholar or Scopus using the following keywords alone or in combinations: “rhegmatogeous retinal detachment”, “proliferative vitreoretinopathy”, “retinotomy”, “retinectomy”, “biomechanics”, “mechanics”, “pathophysiology”, “pathology”, “risk factors” and “treatment”. Manual search from list of bibliography of major articles was also performed to identify key references in this area. Articles were sorted and rated according to relevance and summarized to produce this review.

## Data Availability

Not applicable.
